# Consensus-based statements for assessing clinical competency in podiatry-related work integrated learning

**DOI:** 10.1186/s13047-023-00639-7

**Published:** 2023-07-19

**Authors:** Ryan S. Causby, Sindhrani Dars, Malia Ho, Steven Walmsley, Shannon Munteanu, Helen A. Banwell

**Affiliations:** 1 The University of South Australia, Allied Health & Human Performance Unit, Adelaide, SA 5000 Australia; 2 School of Primary and Allied Health, Monash University, Frankston, VIC 3199 Australia; 3 Division of Podiatry, School of Clinical Sciences, Queensland University of Technology, Brisbane, QLD 4000 Australia; 4 Discipline of Podiatry, School of Allied Health, Human Services and Sport, La Trobe University, Victoria, 3086 Australia

**Keywords:** Clinical competency, Podiatry, Work-integrated learning, Clinical placements, Delphi survey, Consensus

## Abstract

**Background:**

The training of undergraduate and graduate-entry podiatry students in Australia and New Zealand includes practical sessions in a simulated and real-life clinical setting and Work Integrated Learning (WIL) comprising professional clinical placements. Student performance during WIL is evaluated by their Clinical Educators using clinical competency tools. Having a standardised and validated clinical assessment tool for WIL in podiatry would facilitate consistency in assessment, promote standardisation between programs, and ensure that all podiatry students are assessed against a set of criteria over the course of their clinical programs to the point of threshold clinical competency. Therefore, the aim of this study was to develop a series of consensus-based statements via Delphi technique as the first step towards developing guidelines to direct the assessment of podiatry students during WIL.

**Methods:**

This study used a three-round modified Delphi consensus method. A panel of 25 stakeholders was sought. Specifically, representation from each of the universities in Australia and New Zealand who provide entry level programs, Clinical Educators, podiatry student representatives, new podiatry graduates and consumers (podiatrists hiring new graduates). The survey for Round 1 aimed for consensus and consisted of five open-ended questions. Questions one to three asked respondents to nominate what they considered were the important elements that needed to be assessed for podiatry students undertaking WIL for: Clinical performance/skills, Communication and Professional behaviour, Question 4 asked respondents to identify further/other elements of importance, whilst Question 5 asked a) how these elements should be evaluated and b) how should overall competency and ability to progress within the program be determined. Round 2 and 3 aimed to gather agreement and the questions were based on the responses from previous rounds.

**Results:**

Twenty-five participants agreed to participate, 17 females (68%) and eight males (32%). The panel consisted of 10 podiatry educators (40%), nine Clinical Educators (36%), two student representatives (8%), two new podiatry graduates (8%) and two consumers (8%). From the 25 recruited participants, 21 responded to Round one, 18 to Round two and 17 in Round three. At the conclusion of the Delphi survey, 55 statements had reached consensus or agreement.

**Conclusions:**

This Delphi study is the first of its kind for the podiatry profession to develop consensus-based statements regarding the assessment of WIL. Fifty-five statements pertinent to the assessment of WIL were identified. This is an important first step toward the development of a consistent WIL assessment tool which may be applied across entry-level podiatry programs across Australia and New Zealand.

## Background

In Australia, the practice of podiatry is governed by regulatory documents and legislation, namely the National Registration Act [1], Professional Capabilities for Podiatrists [2], and the Accreditation Standards: Entry-level podiatry programs [3]. Entry-level podiatrists need to demonstrate that they possess the professional capabilities to practice podiatry safely and competently within these legislative bounds. The training of undergraduate and graduate-entry podiatry students includes theoretical lessons in a classroom and online setting, practical sessions in a simulated and real-life clinical setting, and Work Integrated Learning (WIL) comprising professional clinical placements. WIL is an important part of learning in the health sciences, as it provides the truest form of contextual learning, whereby learners make meanings by contextualising the content within the learning environment in the workplace [4], as well as incorporating authentic assessment which ensures graduates meet professional competencies and are ‘work-ready’.

*“One of the prime purposes of WIL is to learn through observation what it means to be a professional in the discipline * [5]* and the assessment of professional competence drives this learning” * [6]*.*

Student knowledge and skills are assessed via written assessments, written and practical exams, and Objective Structured Clinical Examinations (OSCEs). Student performance during WIL is evaluated by their Clinical Educators using clinical competency tools.

Clinical competency tools should be able to ensure that students demonstrate professional and ethical behaviour, are good communicators and collaborators, and are competent in practising safely, in accordance with their level of progression in a program. The clinical competency tools can be used in a formative and summative manner. In addition, these tools can be used to allow appropriate reporting of poor performance, concerning behaviour, and track student progress across the course.

At the time of study design and implementation there were nine universities providing podiatry education in Australia and one in New Zealand. Podiatry students’ performance in WIL is assessed based on bespoke clinical competency tools developed in-house by the respective universities. This raises the potential of non-standardised approaches that may not offer consistency in overarching conceptual basis, scaling, reliability, and validation processes. By comparison, standardised assessment of WIL has been developed and widely adopted for other allied health professions, including physiotherapy (Assessment of Physiotherapy Practice) [7], speech pathology (Competency Assessment in Speech Pathology Assessment) [8] and occupational therapy (Student Practice Evaluation Form – Revised Edition) [9].

Having a standardised and validated clinical assessment tool for WIL in podiatry would facilitate consistency in assessment, promote standardisation between programs, and ensure that all podiatry students are assessed against a set of criteria over the course of their clinical programs to the point of threshold clinical competency. Therefore, the aim of this study was to develop a series of consensus-based statements as the first step towards developing guidelines to direct the assessment of podiatry students during WIL. To ensure all voices could be heard equally, with anonymity, a Delphi consensus survey method was employed, seeking broad consultation with stakeholders, including providers, facilitators and end-users (students and consumers) of WIL [10].

## Methods

This study used a three-round modified Delphi consensus method, where key stakeholders and experts in the field were invited to participate in a series of surveys seeking their views of key conceptual elements that underpin competency in clinical practice. As a common method of determining consensus in the absence of guidelines, the Delphi technique allows for a flexible approach to gain large amounts of data [11], with the ability to be conducted online in its entirety. The development and reporting of this study follows the Recommendations for the Conducting and Reporting of Delphi Studies (CREDES) [12]. This study was approved by the University of South Australia’s Human Research Ethics Committee (Protocol number 203578).

### Survey development

A purpose-built survey was developed by the authorship group for Round one in keeping with the novel aims of the study. Round one questions were purposefully open-ended to identify respondents’ individual thoughts and suggestions related to WIL assessment. The questions were initially developed by three of the authors (RC, HB, MH) following review of WIL assessment tools provided by several Australian and New Zealand providers of entry-level podiatry programs who responded to our request (i.e., Auckland University of Technology, Central Queensland University, Charles Sturt University, La Trobe University, Southern Cross University, University of Newcastle, University of South Australia, Western Sydney University). The full authorship group reviewed the questions before implementation of the survey, with wording modified based on their feedback.

The final survey for Round one consisted of five open-ended questions (Appendix 1). Questions one to three asked respondents to nominate what they considered were the important elements that needed to be assessed for podiatry students undertaking WIL for:

1. Clinical performance/skills
2. Communication
3. Professional behaviour

Question 4 asked respondents to identify further/other elements of importance, whilst Question 5 asked a) how these elements should be evaluated (e.g., pass/fail, Likert scale, graded), and b) based on this evaluation approach, how should overall competency and ability to progress within the program be determined.

Rounds two and three of the survey were developed based on comments and responses received in the previous rounds.

### Participants

A panel of 25 stakeholders were sought. The aim of recruitment was to seek a panel that had expertise in delivering WIL (e.g., providers and facilitators), and those with varied experiences of WIL (facilitators and end-users). Specifically, we sought expertise in WIL via academic providers, seeking representation from each of the universities in Australia and New Zealand who provide entry level podiatry programs (*n* = 10). For facilitators with expertise and experience of WIL we sought Clinical Educators (*n* = 9) who have been engaged in supervising and assessing students in WIL. End-users with experience of WIL included podiatry student representatives (*n* = 2), new podiatry graduates (*n* = 2) and consumers (which for the purpose of this study were podiatrists who had employed two or more new graduates within the previous five years) (*n* = 2). Except for student representatives, new graduates and consumers, respondents were required to have a minimum of three years’ experience supervising and assessing students clinically.

As podiatry is a relatively small health profession, and podiatry academia a very small subset, the authorship team took steps to reduce the potential for introduced bias. Recruitment for this study was conducted via email. Emails were disseminated to the Program Leads in the 10 universities in Australia and New Zealand with a podiatry program and Program Leads were asked to nominate potential candidates who they believed met the criteria outlined above. A research assistant (SD) then contacted each nominee directly via email with an information sheet attached, and instructions to respond with a confirmation if they were willing to participate. To minimise location bias, the *a priori* decision was to recruit from a mixture of geographical locations if respondent interest exceeded requirements. This was managed by identifying state of practice of potential respondents and ensuring a representation of states were included (e.g., if our consumers came from Victoria and Queensland, then we first approached the nominated new graduates from Western Australia and New South Wales). To improve the robustness of outcomes, all potential respondents were asked to commit and respond to all three rounds at enrolment, maintain anonymity throughout the study period, keep individual responses confidential and agree to be contacted by email as a method of alerting and reminding the respondents of survey rounds requiring attention. No enticements or compensation were provided, and participants could withdraw their consent of participation at any time during the study period.

### Procedure

Participants who met the inclusion criteria and were included in the study received individual link invitations to each survey round via participant-provided email. Implementation of the Delphi process was undertaken by a research assistant (SD) to minimise the risk of potential conflicts of interest from the authors with participants. All data were collected using the online survey platform SurveyMonkey^©^ (Momentive Inc., California, USA). Respondents confirmed consent at the start of the online survey for Round one, with skip logic engaged to exclude respondents who did not consent. All rounds were open for four calendar weeks and participants were reminded by email one week before the closing date of the survey. Those failing to respond were contacted by email after the closing date and offered a further extension if required. Participants that did not respond to the survey or follow up emails within two-weeks after the closing date were considered non-responders. Participants were supplied a copy of their individual responses each round and supplied a summary of results at the completion of the study where requested.

Participants were able to make comments in Round one and two only. Statements for Round two and three were developed from individual comments made by respondents in the respective preceding rounds. Comments were themed via inductive qualitative content analysis [13], which allows individual comments to be considered, and statements developed on the overarching theme. Further comments were then considered and either deemed consistent with an existing statement or a new statement was developed accordingly. All comments were initially themed by three authors independently (RC, SD and HB), with inconsistencies discussed until agreement. Acknowledging the bias that may occur due to the collegial nature of the authors involved in the analysis (i.e., all three are employed at the same institution), a fourth author (MH) re-coded comments independently with disagreements resolved by discussion.

Statements were accepted if they reached ≥ 70% consensus or agreement. This required 70% or more of the respondents to identify the same themed statement in Round one (consensus) or indicate that they agreed or strongly agreed (on a five-point Likert scale) with a themed statement in Round two or three (agreement). All themed statements from Round one were returned to participants in Round two. Round three included themed statements where 50 to 69% of participants had agreed or where there were additional comments from Round two, to ensure adequate consideration. If less than 50% of participants agreed to a statement it was excluded from future rounds. This percentage of consensus and agreement is consistent with existing and recent literature on the modified Delphi technique [14, 15].

## Results

A total of 45 nominations were received for potential participants. Twenty-five participants agreed to participate, 17 females (68%) and eight males (32%). The panel consisted of 10 academic providers (40%), nine Clinical Educators (36%), two student representatives (8%), two new podiatry graduates (8%) and two consumers (8%). While recruitment met our expertise and experience aims, there was a shortfall in geographical representation. Overall, our panel included three participants from Queensland (12%), nine from New South Wales (36%), three from Victoria (12%), four from South Australia (16%), one from Western Australia (4%) and five from New Zealand (20%). There was no representation from the Northern Territory, Australian Capital Territory or Tasmania.

**Fig. 1:**
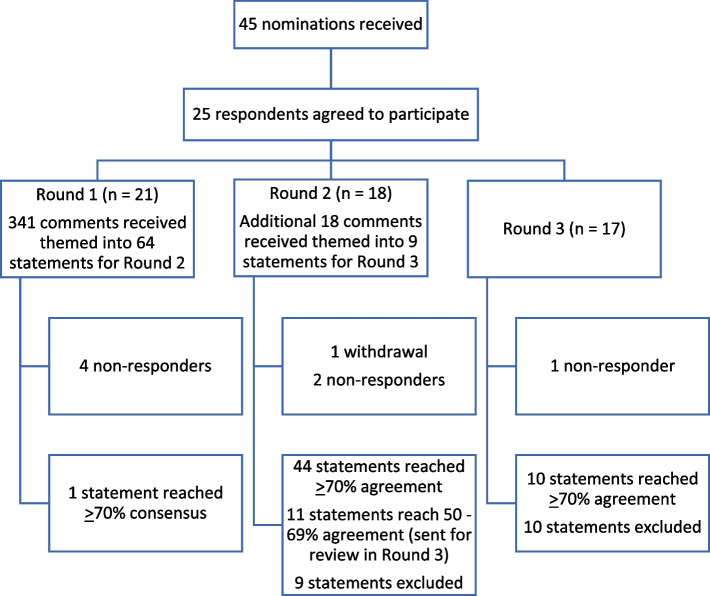
Flow of Delphi survey participants and survey characteristics through the three rounds

From the 25 recruited participants, 21 responded to Round one, 18 to Round two and 17 in Round three. One participant withdrew from the study shortly after Round two had been sent out, the other seven were non-responders. Figure 1 outlines the flow of participants and survey characteristics through the three rounds.

From Round one, 341 comments were received from 21 respondents. Following analysis, one statement met consensus, “Demonstrates safe and effective scalpel skills” (Table 1). Sixty-four further statements were developed based on the comments provided, these statements were returned to respondents to seek agreement for Round two.

**Table 1:** Statements accepted related to assessable elements of importance for podiatry students in work-integrated learning/placements

Category	Statement	Qualifiers and examples	Round accepted	Agreed responses (%)
**Clinical performance/ skills**	Demonstrates safe and effective scalpel skills		One	19/22 (86.4)
	Appropriate identification and implementation of infection control principles and procedures	Hand hygiene, PPE, safe use and disposal of sharps, aseptic techniques, management of waste and blood spills	Two	18/18 (100)
	Maintains appropriate consideration to workplace health and safety	In keeping with the setting requirements (e.g., within clinical or orthotic laboratory settings)	Two	18/18 (100)
	Maintains an evidence-based approach to assessment, diagnosis, management, and education of clients	I.e. demonstrates a knowledge of the evidence base for diagnostic modalities and applies this knowledge to patient treatment	Two	18/18 (100)
	Displays competent and targeted approaches for client assessment	Vascular, neurological, dermatological, biomechanical/anatomical assessment as directed by client presentation and/or student experience	Two	18/18 (100)
	Demonstrates clear and appropriate history taking	Includes recognition/understanding/interpretation of cultural, social, and medical factors, including pharmacological agent use and reports from other health professionals, that may impact health	Two	18/18 (100)
	Displays effective clinical reasoning	In the diagnosis (and differentials) including identifying and reporting of risk factors	Two	18/18 (100)
	Displays expected knowledge of foot and lower limb pathologies	Including their aetiologies and relevant anatomical structures	Two	18/18 (100)
	Uses best-practice guidelines where available	Uses established wound classification tools, conducts appropriate radiology referrals	Two	17/18 (94.4)
	Identifies and develops client focused intervention and/or management plans	Incorporating population-based requirements where relevant (e.g., paediatrics, high risk, culturally diverse groups etc.) and including client education	Two	17/18 (94.4)
	Communicates with and defines client goals	Includes clients in discussions	Two	17/18 (94.4)
	Demonstrates appropriate adaptability	Is able to monitor, evaluate and adapt intervention and management plans as required, including responding to adverse events	Two	17/18 (94.4)
	Demonstrates safe and efficient nail care	Relevant to experience level (e.g., surgical removal of ingrown toenails where relevant)	Two	17/18 (94.4)
	Demonstrates effective biomechanical assessment	Including gait analysis and footwear assessment	Two	17/18 (94.4)
	Works collaboratively	Conducts appropriate referral and/or works appropriately with other health professionals	Two	16/18 (88.9)
	Demonstrates safe and efficient wound management	Including identification and classification of wounds, safe debridement and offloading/dressing/ulcer management techniques	Two	16/18 (88.9)
	Demonstrates safe and competent manufacture and use of simple offloading devices	Such as padding and simple insoles	Two	16/18 (88.9)
	Demonstrates appropriate orthotic prescription (customised or prefabricated)	Appropriate foot morphology capture, manufacture, modification and fitting of devices	Two	16/18 (88.9)
	Ability to use credible sources of information	Electronic Monthly Index of Medical Specialties (eMIMS) or Australian Medicines Handbook (AMH) for pharmaceutical information	Three	15/17 (88.2)
	Competent treatment skills	Including but not limited to: use of exercise prescription, physical therapies, pharmaceuticals and medicaments, footwear, taping	Three	13/17 (76.475)
**Elements of Communication**	Demonstrated note taking abilities	Has the ability to produce legible, accurate, structured and precise clinical documentation in written and/or electronic form incorporating all important information of history, diagnosis, assessment and plan	Two	18/18 (100)
	Obtains consent	For treatment and including financial consent where relevant	Two	18/18 (100)
	Ability to clearly and appropriately communicate	Including questions regarding subjective history and outcomes of consultation, diagnosis, pathology and further investigations or management required with the client, incorporating a client centred care approach	Two	17/18 (94.4)
	Demonstrates culturally appropriate communication	Shows sensitive and safe communication style, considering language, health literacy and any other identified barriers (specifically as it relates to First Nation's Peoples, people with differing physical or cognitive ability, and those from culturally diverse backgrounds)	Two	18/18 (100)
	Ability to develop an appropriate referral letter, report or investigation request	This could be as simple as an x-ray referral or pathology request, up to a detailed referral to an orthopaedic surgeon	Two	18/18 (100)
	Ability to communicate appropriately with people involved in client care	Clear verbal, non-verbal or written communication with family/carers, supervisors, interprofessional teams, admin, peers etc., adapting terminology as required	Two	17/18 (94.4)
	Demonstrates appropriate verbal and/or written communication	An ability to communicate verbal and/or written education specific to clients’ needs	Two	17/18 (94.4)
	Clear setting and communication of client centred goals	Uses a goal setting approach to treatment, created in conjunction with the patient	Two	16/18 (88.9)
	Demonstrated proficient handover	Professional and competent written and verbal clinical handover (ISBAR)	Two	16/18 (88.9)
	An understanding of the legislative requirements of clinical documentation	I.e. confidentiality legislation, note-taking requirements, record retention requirements	Two	15/18 (83.3)
	Proficient in SOAP/E format	Correctly records information in the appropriate format and sections of the medical record	Two	15/18 (83.3)
	Demonstrated competency in rapport building	With patients, patients’ family/carers, colleagues and peers	Three	16/17 (94.1)
	Ability in eliciting client needs	Including conducting motivational interview and identify patient's emotional attachment to the presenting problem	Three	14/17 (82.45)
	An understanding of Telehealth and it's use	Including demonstrated understanding of limitations and appropriate interaction	Three	12/17 (70.6)
	Ability to communicate and participate in a group setting	Service meetings, patient education sessions	Three	15/17 (88.2)
	Demonstrated academic writing/presentations skills	Such as in-servicing for peers	Three	13/17 (76.5)
**Professional behaviour**	Demonstrates professional and appropriate communications	With supervisors, peers and clients (including maintaining boundaries, respectful tone)	Two	17/17 (100)
	Maintains an evidence-based approach to practice	Including critically appraising new evidence	Two	17/17 (100)
	Maintains a professional approach to practice	Including punctual attendance, time management and appropriate clothing and footwear choices (neat, tidy, relevant)	Two	18/18 (100)
	Demonstrates and acts in accordance with relevant legislation, professional standards and guidelines	Consent, infection control, confidentiality, workplace health safety and welfare	Two	18/18 (100)
	Demonstrates sound clinical reasoning	A wholistic understanding of elements and considerations for making a particular diagnosis	Two	18/18 (100)
	Practices in an ethical manner	Acting in the best interests of the patients, together with an understanding of ethical principles	Two	17/18 (94.4)
	Recognises own limitations and seeks assistance as required	This may be achieved through self-reflection or in a clinical context	Two	17/18 (94.4)
	Engages in self learning/development	Adopts self-reflection, seeks feedback, has enthusiasm and willingness for learning	Two	17/18 (94.4)
	Practices in a culturally safe manner	A demonstrated understanding and awareness of cultural safety, including the complex nature of working with translators	Two	16/18 (88.9)
	Maintains a person-centred approach	Specifically a patient-centred approach	Two	15/18 (83.3)
**Other elements**	Interpreting diagnostic reports	Includes X-ray, ultrasound, pathology	Two	17/18 (94.4)
	Ability to practice autonomously	Within their own capabilities and level of experience	Two	16/18 (88.9)
**Further comments**	Some mistakes are expected from students as they are still learning, it is important to provide opportunities to learn from these mistakes	It’s important for students to recognise this as a learning opportunity and accept feedback on this, as well as for supervisors to be understanding of these mistakes	Two	18/18 (100)
	Expectations from clinical supervisors should be in light of resources and patient groups available at placement sites	All experiences and consideration relevant to the presented opportunities	Two	15/18 (83.3)

During Round two, 18 respondents indicated their level of agreement to the returned statements and made 18 further comments. Following analysis, 44 statements met the pre-determined level of ≥ 70% agreement (Table 1) and 11 statements required review in Round three (i.e. had obtained 50 to 69% agreement). Nine new statements were developed based on the comments provided. A total of 20 statements were returned to respondents in Round three.

Round three had 17 respondents, with 10 statements meeting the pre-determined level of ≥ 70% agreement (Fig. 1).

At the conclusion of the Delphi survey, 50 statements relating to assessable elements of importance (Table 1) and five statements relating to grading or evaluation (Table 2) had reached consensus or agreement. Excluded statements that did not meet the minimum 50% agreement required, or were out of the scope of this study, are available in Appendix 2 (Table 3).

**Table 2:** Statements accepted related to grading or evaluation of podiatry students in work-integrated learning/placements

Element	Statement	Round accepted	Agreed responses (%)
**Preferred grading or evaluation**	Pass/Competent on non-graded elements (e.g., competencies, infection control, OHS and professional conduct)	Two	15/18 (83.3)
	Ordinal scale (e.g., 0 to 5, 0 to 100)	Three	13/17 (76.5)
	Option for supervisor to defer judgement if insufficient observations	Three	12/17 (70.6)
**Minimal level of grading**	Pass/Competent for non-graded elements	Two	17/18 (94.4)
	Over the mid-point of a Likert-like scale	Three	15/17 (88.2)

## Discussion

This study obtained consensus-based statements on essential elements when assessing podiatry students’ competency during WIL, as informed by podiatry academics, Clinical Educators, students and end-users. It ensures the necessary first step in the development of a valid WIL assessment tool specific for podiatry students, which will ultimately assist in consistency in clinical assessment across providers of entry-level podiatry programs in Australia and New Zealand.

Based on our findings, the essential elements identified by the Delphi technique share consistency with existing documentation. The primary elements from this study focus on competent clinical skills, communication and professional behaviour. These are consistent with several elements of the Professional Capabilities for Podiatrists document (2022), developed by the Podiatry Accreditation Committee of the Podiatry Board of Australia [2]. The Professional Capabilities document covers five domains of expected competence for registered podiatrists: knowledge and skills; communication and collaboration; professional and ethical practice; lifelong learning; and quality and risk management. Arguably, the only domain not essential to the WIL experience of students is that of ‘lifelong learner’ due to its focus on continued learning and mentorship of peers/other health professionals, which is outside the need or ‘capabilities’ as they relate to students. Encouragingly, even though our findings do not specify a ‘quality and risk’ component, elements relevant to student expectations are covered in ‘Professional behaviour’ (such as, demonstrates and acts in accordance with relevant legislation, professional standards and guidelines, including consent, infection control, confidentiality, workplace health, safety and welfare). Similarly, many of our essential elements reflect those used within the COMPASS tool for speech pathology students [8], the Assessment for Physiotherapy Practice [APP] [7], and the Student Practice Evaluation Form – Revised (Second Edition) [SPEF-R2] (for Occupational therapists) [9]. As one example, our findings indicate students should have the “Ability to communicate appropriately with people involved in client care”, whereas the COMPASS requires students to ‘Communicate effectively with work teams’, the APP requires students to “Communicate effectively and appropriately – verbal/non-verbal”, and the SPEF-R2 has “communicates effectively with service users and significant others” as a core objective.

Another notable finding was that there is evidence to support the main essential elements accepted by our panel. Reynolds and McLean [16], when investigating Clinical Educator perceptions of podiatry students’ placement practice, identified that deficiencies in practical clinical skills and communication abilities contributed to a lack of preparedness. It is potentially this perceived importance of professional and communication skills in clinical performance, where neither are mutually exclusive, that led to several essential elements being identified across categories. For example, ‘Demonstrates clear and appropriate history taking’ in the Performance/Clinical Skills section is similar to ‘Demonstrates note taking abilities’ identified in the Communication section. This speaks to the integrated nature of clinical practice, where it is acknowledged that no singular skill or task in isolation makes a good practitioner.

Of interest, many of the outcomes accepted by respondents relating to clinical skills were often specific. For example, the single consensus statement relates to ‘safe and effective scalpel skills’, whilst statements focused on biomechanical assessment, orthotic manufacture, wound and nail care were also accepted. While these skills are irrefutably important, there were notably some podiatry-related tasks that were not identified or accepted as essential elements for the assessment of WIL activities (e.g., assessment and management of paediatric clients, serial casting for musculoskeletal concerns). Ideally a universal assessment tool needs to be adaptable to a broad range of WIL experiences, able to be applied across cohorts with different levels of experience, adaptable to different levels of competence, and responsive to changing technology and practice scope (such as evolving methods of orthoses manufacture) to maintain relevance. This was identified and incorporated into the key elements by the respondents, with statements requiring students to maintain knowledge and identify client focused, evidence-based, appropriately informed strategies for management of conditions that demonstrate clear clinical reasoning reaching 100% agreement. These elements are essential to ensure the tool remains relevant, ‘future-proof’ and able to be nuanced to individual institutions.

With regards to grading scales, this study found that the preference was for a clear pass grade to determine baseline competency. This can be determined by a giving a pass/fail grade, or a mark over the midpoint of a Likert scale. This is similar to the APP [7] which uses a 5-point Likert scale to grade students’ competencies with the mid-line being the base requirement for success. It must be noted that WIL activities occur at different points depending on the university program structure. Clear guidelines need to be developed to assist Clinical Educators to rate students’ competency according to their progress within the program.

The consensus statements developed in this study represent the initial step to inform the development of a standardised WIL assessment tool. However, the statements may require amalgamation or refinement with the aim of improving brevity and clarity. Further work is required to develop clear assessment criteria, with explanatory notes and examples. However, once developed, this tool may offer entry-level program providers and students greater validity and consistency in assessment of WIL, provide Clinical Educators with more guidance on what is expected of students, and allow accrediting and registration bodies greater confidence that graduating students from different programs have been assessed against the same criteria. Ultimately, this has the potential to help ensure consistency in the clinical capabilities of graduates entering the workforce resulting in improved patient experiences. Any subsequently developed tool may also prove to have international implications where podiatrists train in similar structures to Australia and New Zealand.

There are limitations of this study that need to be considered. All statements required consensus or agreement from the respondents but, in the context of evidence-based practice, represents low-level evidence and expert opinion only. When selecting a manageable number of participants for the study, there was a particular focus on expertise and experience within the Podiatry profession (particularly within the Australian and New Zealand context), however this in itself was a limitation, and the panel may have benefited from experience external to the profession. Further to this, when choosing ‘consumer representation’, we chose to interpret the consumer as the ‘employers’ who then take on the graduates when they complete and enter the real world. It could be argued that the panel may have still benefited from the input of people who receive podiatry care for a particular complaint. Despite transparently supplying respondents with a copy of their comments prior to each round to ensure they were satisfied with our management of them, there is potential that the authorship team could have introduced bias during the theming of statements. The act of theming statements may also, inadvertently, remove detail or nuance from respondents' initial comments. While it is intended that further investigations of the usability of a WIL tool may assist to define or develop statements as needed, it is important to acknowledge that ambiguity may exist in the data as provided within this study. Furthermore, we acknowledge that Round one questions, as created by the authorship group, may have introduced bias given our clear understanding of the current Australian ‘Professional capabilities for podiatrists’ [2] and experience in the assessment of students undertaking WIL. Finally, the strengths of a Delphi technique are enhanced by the anonymity of participants and maintaining confidentiality of responses/respondents. Whilst respondents were asked to maintain anonymity throughout the process, podiatry is a small profession and the chance of intentional or non-intentional collusion of responders cannot be guaranteed.

## Conclusions

This Delphi study is the first of its kind for the podiatry profession to develop consensus-based statements regarding the assessment of WIL. Through broad representation from aspects of providers and facilitators (academics and Clinical Educators), learners (students and new graduates) and stakeholders (employers) 55 statements pertinent to the assessment of WIL were identified. This is an important first step toward the development of a consistent WIL assessment tool which may be applied across entry-level podiatry programs.

## Data Availability

N/A.

## Appendix 2

**Table 3:** Statements excluded for not meeting the required minimum of 50%/70% agreement

Category	Statement	Round excluded	Agreed responses (%)	Reason of exclusion
**Clinical performance/ skills**	Ability to accurately use medical terminology and approved Australian Health Practitioner Regulation Agency (AHPRA) abbreviations in clinical documentation	Three	10/17 (58.8)	< 70% agreement
**Elements of Communication**	An ability to work with interpreters	Two	8/18 (44.4)	< 50% agreement
	An understanding of, and preferably experience in, local (e.g., state-based) placement specific reporting requirements	Three	6/17 (35.3)	< 70% agreement
	The ability to adapt to non-traditional communication methods (e.g., use of Zoom/Teams)	Three	9/17 (52.9)	< 70% agreement
**Professional behaviour**	Use of personal electronic devices such as mobile phones should be limited to break time or if absolutely necessary	Three	11/17 (64.7)	< 70% agreement
**Other elements**	Appropriate use of technology (including social media)	Three	10/17 (58.8)	< 70% agreement
	Ability to contribute to broader service needs: (e.g., participating in quality improvement, health promotion, admin, marketing, supervision at an appropriate level)	Three	11/17 (64.7)	< 70% agreement
**Preferred grading or evaluation**	Likert scale	Two	7/18 (38.8)	< 50% agreement
	Use of a rubric	Three	9/13 (69.2)	< 70% agreement
	Interval scale for formative and non-graded pass/fail for summative assessments	Three	9/17 (52.9)	< 70% agreement
**Minimal level of grading**	Mid-point of a Likert scale	Two	4/17 (23.5)	< 50% agreement
	100% of interval scale	Two	1/18 (5.6)	< 50% agreement
	70 to 80% of interval scale	Two	4/18 (22.2)	< 50% agreement
	60% of interval scale	Two	7/18 (38.9)	< 50% agreement
	50% of interval scale	Two	4/17 (23.5)	< 50% agreement
**Further comments**	Three strikes for safety/infection control and then fail	Two	6/18 (33.3)	< 50% agreement
	It would be beneficial to have consistent and standardised definitions and assessment tools to be utilised across different sectors and states	Two	-	Out of Scope of this study
	Two strikes for un-professional behaviour and then fail	Three	9/17 (52.9)	< 70% agreement
	Incorporation of tools like ‘Learning-styles questionnaires’ to help supervisors with best ways to support student learning	Three	9/17 (52.9)	< 70% agreement

*AHPRA* Australian Health Practitioner Regulation Agency (AHPRA)

## Appendix 3

**Table 4:** Final statements accepted for the assessment of podiatry students in work-integrated learning/placements

Category	Statement
**Clinical performance/ skills**	Demonstrates safe and effective scalpel skills
	Appropriate identification and implementation of infection control principles and procedures
	Maintains appropriate consideration to workplace health and safety
	Maintains an evidence-based approach to assessment, diagnosis, management, and education of clients
	Displays competent and targeted approaches for client assessment
	Demonstrates clear and appropriate history taking
	Displays effective clinical reasoning
	Displays expected knowledge of foot and lower limb pathologies
	Uses best-practice guidelines where available
	Identifies and develops client focused intervention and/or management plans
	Communicates with and defines client goals
	Demonstrates appropriate adaptability
	Demonstrates safe and efficient nail care
	Demonstrates effective biomechanical assessment
	Works collaboratively
	Demonstrates safe and efficient wound management
	Demonstrates safe and competent manufacture and use of simple offloading devices
	Demonstrates appropriate orthotic prescription (customised or prefabricated)
	Ability to use credible sources of information
	Competent treatment skills
**Elements of Communication**	Demonstrated note taking abilities
	Obtains consent
	Ability to clearly and appropriately communicate
	Demonstrates culturally appropriate communication
	Ability to develop an appropriate referral letter, report or investigation request
	Ability to communicate appropriately with people involved in client care
	Demonstrates appropriate verbal and/or written communication
	Clear setting and communication of client centred goals
	Demonstrated proficient handover
	An understanding of the legislative requirements of clinical documentation
	Proficient in SOAP/E format
	Demonstrated competency in rapport building
	Ability in eliciting client needs
	An understanding of Telehealth and it's use
	Ability to communicate and participate in a group setting
	Demonstrated academic writing/presentations skills
**Professional behaviour**	Demonstrates professional and appropriate communications
	Maintains an evidence-based approach to practice
	Maintains a professional approach to practice
	Demonstrates and acts in accordance with relevant legislation, professional standards and guidelines
	Demonstrates sound clinical reasoning
	Practices in an ethical manner
	Recognises own limitations and seeks assistance as required
	Engages in self learning/development
	Practices in a culturally safe manner
	Maintains a person-centred approach
**Other elements**	Interpreting diagnostic reports
	Ability to practice autonomously

## Appendix 4

**Table 5:** Recommendations for the Conducting and REporting of DElphi Studies (CREDES) [12]

Items of reporting	Reported on page
*Purpose and rationale.* The purpose of the study should be clearly defined and demonstrate the appropriateness of the use of the Delphi technique as a method to achieve the research aim. A rationale for the choice of the Delphi technique as the most suitable method needs to be provided	Background, Page 3 & 4
*Expert panel.* Criteria for the selection of experts and transparent information on recruitment of the expert panel, sociodemographic details including information on expertise regarding the topic in question, (non)response and response rates over the ongoing iterations should be reported	Methods, Page 6
*Description of the methods.* The methods employed need to be comprehensible; this includes information on preparatory steps (How was available evidence on the topic in question synthesised?), piloting of material and survey instruments, design of the survey instrument(s), the number and design of survey rounds, methods of data analysis, processing and synthesis of experts’ responses to inform the subsequent survey round and methodological decisions taken by the research team throughout the process	Methods, page 5 – 8
*Procedure.* Flow chart to illustrate the stages of the Delphi process, including a preparatory phase, the actual ‘Delphi rounds’, interim steps of data processing and analysis, and concluding steps	Results, page 10
*Definition and attainment of consensus.* It needs to be comprehensible to the reader how consensus was achieved throughout the process, including strategies to deal with non-consensus	Methods, page 8
*Results*. Reporting of results for each round separately is highly advisable in order to make the evolving of consensus over the rounds transparent. This includes figures showing the average group response, changes between rounds, as well as any modifications of the survey instrument such as deletion, addition or modification of survey items based on previous rounds	Results and Table 1, Pages 10—18
*Discussion of limitations.* Reporting should include a critical reflection of potential limitations and their impact of the resulting guidance	Discussion pages 19 – 20
*Adequacy of conclusions.* The conclusions should adequately reflect the outcomes of the Delphi study with a view to the scope and applicability of the resulting practice guidance	Discussion and conclusion, page 19 – 20
*Publication and dissemination*. The resulting guidance on good practice in palliative care should be clearly identifiable from the publication, including recommendations for transfer into practice and implementation. If the publication does not allow for a detailed presentation of either the resulting practice guidance or the methodological features of the applied Delphi technique, or both, reference to a more detailed presentation elsewhere should be made (e.g. availability of the full guideline from the authors or online; publication of a separate paper reporting on methodological details and particularities of the process (e.g. persistent disagreement and controversy on certain issues)). A dissemination plan should include endorsement of the guidance by professional associations and health care authorities to facilitate implementation	Discussion Page 19 part and N/A
